# Design of a stand-alone hybrid dispersed generation network unified by passivity-based control

**DOI:** 10.1098/rsos.230458

**Published:** 2024-07-10

**Authors:** Rutvika Manohar, Takashi Hikihara

**Affiliations:** ^1^ Graduate School of Engineering, Kyoto University, Katsura, Kyoto 615-8510, Japan

**Keywords:** passivity-based control, nonlinear control, DC/AC microgrids

## Abstract

In this article, we propose a model for a stand-alone hybrid distributed generation system. In this model, the input sources are distributed DC sources like solar panels or batteries. The idea behind this network framework is to introduce a hybrid DC/AC network, feasible for small and remotely located areas with stand-alone DC grids, in the vicinity of larger towns requiring a functional AC connection. The behaviour of the system in the steady state is analysed, and the network is mathematically represented with port-controlled Hamiltonian modelling. Stabilization to the desired voltage, both AC as well as DC, is attained with nonlinear passivity-based control taking into consideration not only the energy characteristics but also the inherent physical structure.

## 1. Introduction

Dispersed generation is defined as the decentralized generation of electricity through small generators located near the end-user or the consumer [1,2]. With the global push to move towards renewable sources of energy, the generation of electricity is moved away from centralized power plants and towards individual generator units scattered over towns and villages [3]. This is referred to as distributed or dispersed generation. Distributed generation has been discussed widely as an alternative to solve the electrification problem of rural and remote areas by constructing small stand-alone power grids by installing renewable energy generators in multiple households [4]. Such projects have been developing under the umbrellas of local governments as well as in the private sector [5–8]. Of late, several ideas for stand-alone renewable energy grids have been proposed for rural areas [9–12].

The concept of an AC/DC hybrid microgrid has been discussed in previous works [13–15]. Unlike conventional AC microgrids, an AC/DC microgrid combines the advantages of both the AC and DC microgrids. In this article, we introduce an AC/DC hybrid stand-alone hybrid microgrid with DC and AC subgrids aimed at employing solar panels or other DC sources as the primary inputs. The idea behind this network framework is to introduce a hybrid DC/AC network feasible for small, remotely located areas with stand-alone DC grids, in the vicinity of larger towns requiring a functional AC connection. Towards the goal of rural electrification, stand-alone microgrids with DC inputs such as solar cells have been subsidized by governments in various countries [16,17].

Numerous control methods and power-sharing techniques have been discussed with the above-mentioned AC/DC hybrid microgrid strategies [14,15]. Going beyond regular droop control techniques and linear control typically employed for the control of hybrid microgrids, we aim to step further and provide a control strategy to achieve output voltage control for all the AC and DC subgrids through nonlinear passivity-based control (PBC). PBC was introduced by Popov & Georgescu [18] in the context of electrical circuits. PBC is founded on the basic concept of using the potential and kinetic energy of the system as a cost function to achieve fine tuned control of a desired variable. The strength of PBC comes from the fact that it is possible to guarantee the stability of the entire system by using a control function obtained from the energy function, which in turn is derived from the ‘passivity’ of the system. In this article, we aim at using the nonlinear PBC framework to implement an output voltage control for the DC as well as the AC grids of the hybrid microgrid, as well as frequency control for the AC grid. Thus, by using nonlinear PBC, the microgrid network can have grid-forming capabilities with voltage and frequency control.

The first step of implementing the nonlinear PBC is to develop a design framework that includes the circuit configurations for the hybrid AC/DC microgrid. This design framework refers to port-controlled Hamiltonian modelling (PCHM) and takes into consideration the physical circuit configurations of the entire microgrid including the AC as well as the DC microgrids and the inverters connecting them. The PCHM framework models the potential and kinetic energy functions of the microgrid through the inductances and capacitances while also incorporating the non-energy elements of the circuit such as resistors, diodes and power electronic switches [19,20]. The design framework is also scalable, making it flexible to the incorporation of additional dispersed generation units. The utility of this framework is analysed by developing a control strategy based on the energy characteristics of the entire microgrid. In the context of a hybrid network, consideration has to be given to coupled converters as well as inverters operating concurrently. In this article, we define an energy function such that its minimum corresponds to the desired equilibrium of the entire network. The energy transfer between individual converter/inverter units in a particular network, as well as that between the networks themselves, governs the dynamic behaviour of the hybrid dispersed generation network. As stability, along with scalability in the modelling have been two important questions for microgrid research, PBC has been studied in the context of microgrids. Particularly, DC microgrids have been the main focus of researchers employing PBC techniques owing to the well-understood PBC control for DC/DC converters [21]. Voltage regulation of DC microgrids with variations of PBC has been studied and implemented recently [22–24]. For example, the application of adaptive PBC has been studied in [25], and robust and decentralized PBC for DC microgrids has been studied in [25]. Injection and damping assignment PBC for DC microgrids has been explored in [26]. The literature for the development of PBC for inverters and AC microgrids is relatively scant with [27] proposing to control an islanded AC microgrid with coordinated PBC strategy and a phase synchronization strategy proposed in [28]. For hybrid AC/DC microgrids, Amirkhan *et al*. and Azimi & Hamzeh [29,30] provide two different PBC strategies to be applied to AC/DC hybrid microgrids. To the best of our knowledge, the methods discussed above usually consider generalized linear models, and a nonlinear but computationally light model has not been discussed. Amirkhan *et al*. [29] suggest a PBC focusing on the coordination between AC and DC microgrids, describing in detail the design structure and passivity equations of all the different components in the given AC/DC microgrids. However, a scalable network framework for increasing the number of distributed generation units has not been discussed. Also, the phase synchronization of all the distributed generation units and a steady-state analysis of the inverters has not been provided.

Considering the available literature, the main contributions that highlight the novelty of this work are as follows. (i) *A general circuit framework*: a circuit framework has been provided considering DC/DC boost converters and buck-type inverters as an example for modelling both AC and DC networks in a stand-alone microgrid. As the circuit framework is common to all power electronic converters, it can be used for other types of converter and inverter topologies. To comprehend the scalability of the network, a graph representation is provided with a degree centrality analysis. (ii) *Scalable PCHM*: based on the converters and inverters in the circuit, a matrix framework can be obtained based on the port-controlled Hamiltonian framework. PCHM provides a state matrix with the currents flowing through the inductive elements and the voltage across the capacitive elements, a structure matrix relating to the circuit topology and a dissipation matrix separating the resistive loads in the model. (iii) *Nonlinear PBC for voltage control*: based on the PCHM, an energy function incorporating the potential and kinetic energies of the system can be derived. After proving exponentially asymptotic stability for the given energy function, control equations can be derived to obtain voltage control for the DC as well as AC output voltages. (iv) *Validation*: validation of the control method is provided with simulation results.

## 2. Network formulation

**Figure 1.**
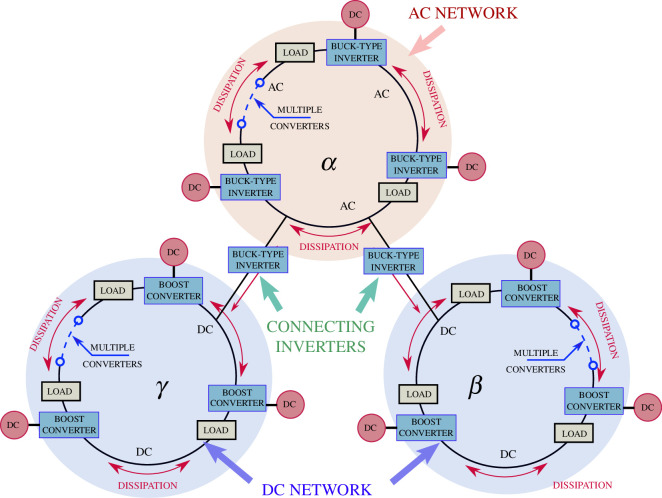
Network configuration.

The structure of the proposed hybrid DC/AC network is described in figure 1. The network configuration shows multiple connected rings, with the α ring comprising multiple DC inputs accompanied by buck-type inverters to obtain a single-phase AC output. The α ring is connected to multiple DC rings ( β and γ ), with DC inputs, which are conditioned with multiple DC/DC boost converters.

Various studies point out that the main challenge in the implementation of a hybrid microgrid with DC/AC subgrids is to manage the power flow between the subgrids by designing an effective method of control. This article discusses the control of the power flow between the subgrids with a control strategy that relies on the energy-conserving structure of the entire network.

Prior to the application of PBC, the system is mathematically formulated using PCHM [31]. As opposed to the traditional Euler–Lagrange modelling, the PCHM framework successfully modifies the potential as well as the kinetic energy functions of the network, thus incorporating non-energy elements such as resistors, diodes and power electronic switches [19,20]. This article presents the successful application of PCHM and PBC to achieve stabilization of the entire network through numerical simulations.

**Figure 2.**
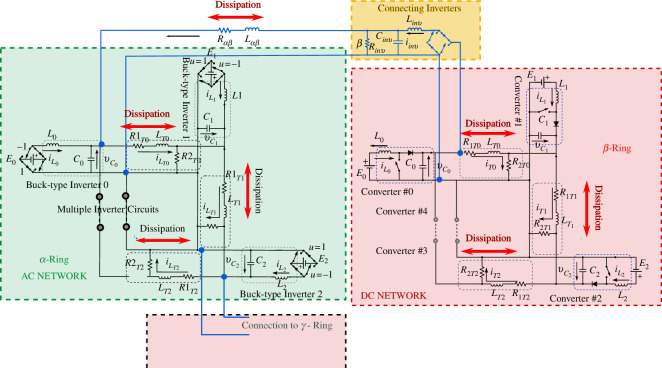
Schematic diagram of connection of AC and DC rings.

Figure 2 shows a schematic diagram for the connection of the α – β rings. The connection of the α – γ rings is assumed to be similar. The coupled converter/inverter units in the ring maintain a constant voltage in the ring through control action. Here, the load resistances ( $R_{2T}$ ) are placed across the capacitor output voltages. Distributed generation implies that each generation unit, i.e. the input (PV) and the power electronic circuitry of the inverters and converters, be dispersed in space. The dissipation elements of line inductor ( $L_{t}$ ) and line resistance ( $R_{1T}$ ) are in series, a model closely resembling the transmission line model. Details of the individual ring configuration can be found in [32]. The number of converters in the ring as well as the number of DC rings connected to the main AC ring ( α ) can be chosen as per user definition, and does not cause any loss of generality.

The system equations are given below and are divided into three parts. First, for the α ring, subscription n denotes the #n inverter in the ring. The equations for the α ring are given in equations (2.1)–(2.3). The control task is to regulate the average output voltage for all the converters/inverters. As the output voltages are either DC or AC with a frequency (usually 50 or 60 Hz) much lower than the switching frequency of the power electronic switches, it is desirable to consider the averaged circuit variables rather than the instantaneous ones. It is assumed that the ripples and harmonics are sufficiently small. Then, the instantaneous switch position will be replaced by a modulating function μ . The averaged circuit variables will be replaced by their instantaneous values.


(2.1) $$L_{\alpha \text{n}}\frac{di_{L\alpha \text{n}}}{dt}=-v_{C\alpha \text{n}}+\mu_{\alpha \text{n}}E_{\alpha \text{n}},$$

(2.2) $$C_{\alpha \text{n}}\frac{dv_{C\alpha \text{n}}}{dt}=i_{L\alpha \text{n}}-i_{T\alpha \text{n}}+i_{T\alpha \text{n}-\text{1}}-\frac{v_{C_{\alpha \text{n}}}}{R_{2\alpha \text{n-1}}}+\eta i_{\alpha \beta }+\zeta i_{\alpha \gamma },$$

(2.3) $$L_{T\alpha \text{n}}\frac{di_{T\alpha \text{n}}}{dt}=v_{C_{\alpha \text{n}}}-v_{C_{\alpha \text{n}+1}}-R_{1\alpha \text{n}}i_{T\alpha \text{n}}.$$

Here, η=1 for the inverter #n of which the output is connected to the DC ring β and ζ=1 for the inverter connected to the DC ring γ . $i_{\alpha \beta }$ and $i_{\alpha \gamma }$ are the currents flowing in the dissipation between the α – β rings and α – γ rings, respectively.

Equations for the connecting inverter between the rings α and β and the dissipation current between these rings are given in equations (2.4)–(2.6):


(2.4) $$L_{\text{inv}\beta }\frac{di_{\text{inv}\beta }}{dt}=-v_{\text{inv}\beta }+\mu_{\text{inv}\beta }v_{C\beta \text{q}},$$

(2.5) $$C_{\text{inv}\beta }\frac{dv_{\text{inv}\beta }}{dt}=i_{\text{inv}\beta }-\frac{v_{\text{inv}\beta }}{R_{\text{inv}\beta }}-i_{\alpha \beta },$$

(2.6) $$L_{\alpha \beta }\frac{di_{\alpha \beta }}{dt}=v_{\text{inv}\beta }-v_{C\alpha \eta }-R_{\alpha \beta }i_{\alpha \beta }.$$

Here #q is the converter where the DC ring β is connected to the AC ring α . The equations for the inverter connecting the α – γ rings assume a similar form. Let #s be the converter of the DC ring γ connected to the α ring.

For the DC Rings β and γ , the format of the equations is the same. Equations (2.7)–(2.9) give the system equations for the β ring. The subscription p denotes the #p converter in the β ring:


(2.7) $$L_{\beta \text{p}}\frac{di_{L\beta \text{p}}}{dt}=\left(1-u_{\beta \text{p}}\right)v_{C\beta \text{p}}+E_{\beta \text{p}},$$

(2.8) $$C_{\beta \text{p}}\frac{v_{C\beta \text{p}}}{dt}=\left(1-u_{\beta \text{p}}\right)i_{\beta \text{p}}-i_{T\beta \text{p}}+i_{T\beta \left(\text{p}-1\right)}-\frac{v_{C\beta \text{p}}}{R_{2T\beta \text{p}}}-\nu \mu_{\text{inv}\beta }i_{\text{inv}\beta },$$

(2.9) $$L_{T\beta \text{p}}{\overset{˙}{i}}_{T\beta \text{p}}=v_{C\beta \text{p}}-v_{C\beta \left(\text{p}+1\right)}-R_{1T\beta \text{p}}i_{T\beta \text{p}}.$$

Here, ν=1 for the converter #p , which is connected to the AC ring α .

## 3. Open-loop system analysis

Before the application of the nonlinear control, the system is analysed as an open-loop system with no feedback control on the duty ratio of the power converters. For the DC rings, it is a constant signal corresponding to the desired output voltage. For the AC rings, and the connecting inverters, however, the duty ratio has to be formulated by solving the differential equation as the derivatives of the state variables are not stable. The results with the application of the open-loop system are helpful to compare the eventual application of the feedback control.

### 3.1. Open-loop system analysis for DC rings

The desired steady state of the DC outputs is constant; thus, the derivative of the state variables becomes zero. Thus, the steady-state variables for the β ring are given as follows:


(3.1) $$\begin{array}{c} {\bar{i}}_{L\beta \text{p}}=\frac{E_{\beta \text{p}}}{R_{2T_{\beta \text{p}-1}}{\left(1-U_{\beta \text{p}}\right)}^{2}}+\frac{1}{R_{1T_{\beta \text{p}}}}\left[\frac{E_{\beta \text{p}}}{{\left(1-U_{\beta \text{p}}\right)}^{2}}-\frac{E_{\beta \text{p}+1}}{\left(1-U_{\beta \text{p}+1}\right)\left(1-U_{\beta \text{p}}\right)}\right]\\ -\frac{1}{R_{1T_{\beta \text{p}-1}}}\left[\frac{E_{\beta \text{p}-1}}{\left(1-U_{\beta \text{p}-1}\right)\left(1-U_{\beta \text{p}}\right)}-\frac{E_{\beta \text{p}}}{{\left(1-U_{\beta \text{p}}\right)}^{2}}\right], \end{array}$$

(3.2) $${\bar{v}}_{C\beta \text{p}}=\frac{E_{\beta \text{p}}}{\left(1-U_{\beta \text{p}}\right)},$$

(3.3) $${\bar{i}}_{T_{\beta \text{p}}}=\frac{1}{R_{1T_{\beta \text{p}}}}\left[\frac{E_{\beta \text{p}}}{\left(1-U_{\beta \text{p}}\right)}-\frac{E_{\beta \text{p}+1}}{\left(1-U_{\beta \text{p}+1}\right)}\right].$$

### 3.2. Open-loop system analysis for AC ring

For the # n buck-type inverter in the α ring, the desired voltage is an AC waveform in the form ${\bar{v}}_{C\alpha \text{n}}=A_{\alpha \text{n}}\sin(2\pi f_{\alpha \text{n}}t)$ . Here, the angular frequency $\omega_{\alpha \text{n}}=2\pi f_{\alpha \text{n}}$ .

Equations (2.1)–(2.3) give the mathematical description of the system for any # n buck-type inverter system. Steady-state analysis is employed to formulate the equilibrium values for the desired inductor current ( ${\bar{i}}_{L\alpha \text{n}}$ ) and the desired dissipation current ( ${\bar{i}}_{T\alpha \text{n}}$ ) for a given desired voltage $v_{C\alpha \text{n}}={\bar{v}}_{C\alpha \text{n}}$ . Then, it will be possible to formulate the sinusoidal duty ratio $U_{\alpha \text{n}}$ .

It is possible to analytically solve the linear differential equation (2.3), by employing the known values of $v_{C\alpha \text{n}}={\bar{v}}_{C\alpha \text{n}}$ , $v_{C\alpha \text{n+1}}={\bar{v}}_{C\alpha \text{n+1}}$ . The solution is given in equation (3.4):


(3.4) $$\begin{array}{c} {\bar{i}}_{T\alpha \text{n}}=\frac{R_{1\alpha \text{n}}A_{\alpha n}}{R_{1\alpha \text{n}}^{2}+L_{T\alpha \text{n}}^{2}\omega_{d\alpha \text{n}}}\left(\sin\left(\omega_{\alpha \text{n}}t\right)-\frac{\omega_{\alpha \text{n}}L_{T\alpha \text{n}}}{R_{1\alpha \text{n}}}\cos\left(\omega_{\alpha \text{n}}t\right)\right)+\frac{R_{1\alpha \text{n}}A_{\alpha \text{n+1}}}{R_{1\alpha \text{n}}^{2}+L_{T\alpha \text{n}}^{2}\omega_{\alpha \text{n+1}}}\left(\sin\left(\omega_{\alpha \text{n+1}}t\right)\;-\frac{\omega_{\alpha \text{n+1}}L_{T\alpha \text{n}}}{R_{1\alpha \text{n}}}\cos\left(\omega_{\alpha \text{n+1}}t\right)\right)+k_{\alpha }e^{-\left(R_{1\alpha \text{n}}/L_{\alpha \text{n}}\right)t}. \end{array}$$

Here, $k_{\alpha }$ is the constant of integration. The value of $k_{\alpha }$ is obtained by applying the initial condition $i_{Td}(0)=0$ :


(3.5) $$k_{\alpha }=\frac{A_{\alpha \text{n}}\omega_{\alpha \text{n}}L_{T\alpha \text{n}}}{R_{1\alpha \text{n}}^{2}+L_{\alpha \text{n}}^{2}\omega_{\alpha \text{n}}}-\frac{A_{\alpha \text{n+1}}\omega_{\alpha \text{n+1}}L_{T\alpha \text{n}}}{R_{1\alpha \text{n}}^{2}+L_{\alpha \text{n}}^{2}\omega_{\alpha \text{n+1}}}.$$

Then, the equilibrium value of $i_{L\alpha \text{n}}$ can be obtained by solving equation (2.1) with $i_{T\alpha \text{n}}={\bar{i}}_{T\alpha \text{n}}$ :


(3.6) $${\bar{i}}_{L\alpha \text{n}}=C_{\alpha \text{n}}{\overset{˙}{\bar{v}}}_{C\alpha \text{n}}-{\bar{i}}_{T\alpha \text{n}}+{\bar{i}}_{T\alpha \text{n}-1}+\frac{{\bar{v}}_{C\alpha \text{n}}}{R_{2\alpha \text{n}-1}}+\eta {\bar{i}}_{\alpha \beta }+\nu {\bar{i}}_{\alpha \gamma }.$$

Finally, the open-loop duty ratio $U_{\alpha \text{n}}$ is obtained from equation (2.2) by substituting $i_{L\alpha \text{n}}={\bar{i}}_{L\alpha \text{n}}$ and given in equation (3.11):


(3.7) $$U_{\alpha \text{n}}=\frac{L_{\alpha \text{n}}}{E_{\alpha \text{n}}}\left[C_{\alpha \text{n}}{\overset{¨}{\bar{v}}}_{C\alpha \text{n}}-{\overset{˙}{\bar{i}}}_{T\alpha \text{n}}+{\overset{˙}{\bar{i}}}_{T\alpha \text{n}-1}+\frac{{\overset{˙}{\bar{v}}}_{C\alpha \text{n}}}{R_{2\alpha \text{n}-1}}\right]+\frac{{\bar{v}}_{C\alpha \text{n}}}{E_{\text{n}}}+\eta {\overset{¨}{\bar{i}}}_{\alpha \beta }+\nu {\overset{¨}{\bar{i}}}_{\alpha \gamma }.$$

### 3.3. Open-loop system analysis for connecting inverters

As the setting for the AC ring, the steady-state analysis can be applied to the inverters between the rings. The desired voltage is set to be the same as the instantaneous desired voltage of the AC ring: $v_{\text{inv}\beta }=A_{\beta }\sin(\omega_{\beta }t)$ . Then, by analytically solving equation (2.6), we can get the following.


(3.8) $$\begin{array}{c} {\bar{i}}_{\alpha \beta }=\frac{R_{\alpha \beta }A_{\beta }}{R_{\alpha \beta }^{2}+L_{\alpha \beta }^{2}\omega_{\beta }}\left(\sin\left(\omega_{\beta }t\right)-\frac{\omega_{\beta }L_{\alpha \beta }}{R_{\alpha \beta }}\cos\left(\omega_{\beta }t\right)\right)-\frac{R_{\alpha \beta }A_{\alpha 0}}{R_{\alpha \beta }^{2}+L_{\alpha \beta }^{2}\omega_{\alpha 0}}\left(\sin\left(\omega_{\alpha 0}\right)\;-\frac{\omega_{\alpha 0}L_{\alpha \beta }}{R_{\alpha \beta }}\cos\left(\omega_{\alpha 0}\right)\right)+k_{\alpha \beta }e^{-\left(R_{\alpha \beta }/L_{\alpha \beta }\right)t}, \end{array}$$

(3.9) $$k_{\alpha \beta }=\frac{A_{\beta }\omega_{\beta }L_{\alpha \beta }}{R_{\alpha \beta }^{2}+L_{\alpha \beta }^{2}\omega_{\beta }}-\frac{A_{\alpha 0}\omega_{\alpha 0}L_{\alpha \beta }}{R_{\alpha \beta }^{2}+L_{\alpha \beta }^{2}\omega_{\alpha 0}}.$$

The other state variables are given as follows:


(3.10) $${\bar{i}}_{\text{inv}\beta }=C_{\alpha \beta }{\overset{˙}{\bar{v}}}_{\text{inv}\beta }-{\bar{i}}_{\alpha \beta }+\frac{{\bar{v}}_{\text{inv}\beta }}{R_{\text{inv}\beta }},$$

(3.11) $$U_{\alpha \beta }=\frac{L_{\alpha \beta }}{{\bar{v}}_{C\beta \text{q}}}\left[C_{\alpha \beta }{\overset{¨}{\bar{v}}}_{\text{inv}\beta }+{\overset{˙}{\bar{i}}}_{\alpha \beta }+\frac{{\overset{˙}{\bar{v}}}_{\text{inv}\beta }}{R_{\text{inv}\beta }}\right]+\frac{{\bar{v}}_{\text{inv}\beta }}{{\bar{v}}_{C\beta \text{q}}}.$$

## 4. Port-controlled Hamiltonian modelling

PCHM is a method to represent systems through the interaction they have with their environment [33]. Application of PCHM to ring coupled converters is given in [28,32], where it is used to model not just rings of DC/DC converters but DC/AC inverters as well and allows for the inclusion of non-energy elements like power electronic switches and load resistances. PCHM is thus the first step in the application of PBC.

PCHM organizes the system into interconnection (**J**), dissipation (**R**) and external input (**E**) matrices within a state space framework [19]. The system model using the port-controlled Hamiltonian (PCH) framework for the hybrid AC/DC system is given in equation (4.2) as in the form given in [34]:


(4.1) $$D\overset{˙}{x}\left(t\right)=\left[J-R\right]\frac{\partial H}{\partial x}+G\left(x\right)E,$$

(4.2) $$y=G^{T}\left(x\right)\frac{\partial H}{\partial x}.$$

For total m inverters/converters in the ring network, x , the state of the system, is a ((m×3)×1) column matrix of all the inductance currents and capacitance voltages. D is a diagonal matrix of the capacitance and inductance of the currents and voltages in the state space configuration, respectively, and represents the coefficients of all the energy elements in the system. The structure matrix, J , is a ((m×3)×(m×3)) skew-symmetric matrix. As the input voltage to the connecting inverters is the output voltage of inverter #q of ring β and converter #r of ring γ , the structure matrix J is a function of $\mu_{\text{inv}\beta }$ and $\mu_{\text{inv}\gamma }$ as well as of the DC/DC converter duty ratios $\mu_{\beta \text{p}}$ and $\mu_{\gamma \text{s}}$ . A ((m×3)×(m×3)) diagonal matrix, G , is a function of $\mu_{\alpha \text{n}}$ . J and G are determined from the structure as per Kirchoff’s law. E is a ((m×3)×1) matrix of the external DC inputs to the corresponding inverters. R , the dissipation matrix, is a ((m×3)×(m×3)) diagonal matrix, with resistance elements. Finally, H is the Hamiltonian of the system which corresponds to the energy of the system.

**Figure 3.**
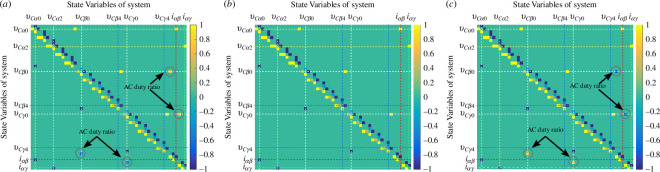
Structure matrix as a function of time: (*a*) $\mu_{\text{inv}\beta }$ , $\mu_{\text{inv}\gamma }=-0.7$ ; (*b*) $\mu_{\text{inv}\beta }$ , $\mu_{\text{inv}\gamma }=0$ ; (*c*) $\mu_{\text{inv}\beta }$ , $\mu_{\text{inv}\gamma }=0.7$ .

As the structure matrix J is a function of the sinusoidal duty ratio of the inverters, it is a function of time. The structure matrix represents the connection of all the state variables. It indicates whether a pair of state variables are connected to each other. Figure 3 gives a graphical representation of the structure matrix of the proposed distributed generation system. It visualizes the structure of the matrix. Here, $\mu_{\beta \text{p}}$ and $\mu_{\gamma \text{s}}$ = 0.6. As $\mu_{\text{inv}\beta }$ and $\mu_{\text{inv}\gamma }$ are sinusoidal, the structure matrix is shown for three values of $\mu_{\text{inv}\beta }$ and $\mu_{\text{inv}\gamma }$ .

**Figure 4.**
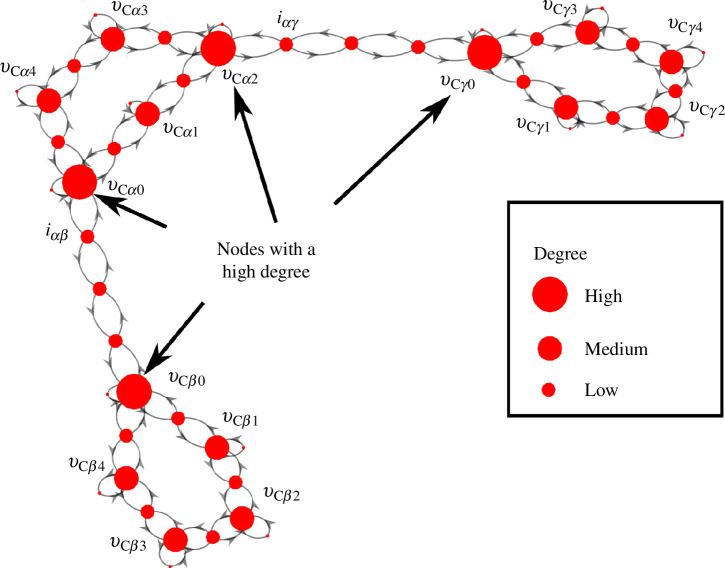
Graph representation with degree centrality analysis.

As the proposed network comprises multiple converter/inverter units, it is desirable to know the connection properties to identify the most important units of the entire network. For a smaller system with fewer number of units, the important units are evident, but for larger systems, not necessarily in ring coupled formulation, such analysis is important to figure out the weak areas of the network. For the proposed AC/DC dispersed generation system, the centrality of the network is shown in the graph representation shown in figure 4. Here, the state variables are represented as nodes of the graph. The size of the nodes is proportional to the degree centrality of the node, indicating that the biggest nodes are the ones with the highest degree. Failure of the higher-degree nodes is more likely to result in the failure of the network as a whole [35]. Thus, this information helps to make the network more robust by safeguarding the vulnerable nodes [36].

## 5. Passivity-based control

The application of PBC to the dispersed generation system with AC and DC voltage output is investigated in this section. PBC can be applied by specifying a desired energy function such that the minimum of this function is the desired equilibrium. The desired energy function, which can be shown to be a candidate for Lyapunov functions, is designed based on the Hamiltonian of the system. The basics of the application of PBC to a passive circuit, especially applicable to ring-connected DC/DC converters or DC/AC inverters is given in [28,32]. It can be shown that the control equation obtained for PBC is given by taking a desired energy function as a candidate of the Lyapunov function with the error function as $e=x-x_{d}$ , where $x_{d}$ is the desired trajectory of the state, the control rule with nonlinear PBC can be formulated for the entire network as given below [28,32].


(5.1) $$D{\overset{˙}{x}}_{d}\left(t\right)=\left(J-R\right)x_{d}\left(t\right)+E+R_{I}\left(x-x_{d}\left(t\right)\right).$$

Here, the damping injection $R_{I}$ is a ((m×3)×(m×3)) diagonal matrix, with damping added to the corresponding inductor current term, making the system asymptotically stable if $R+R_{I}>0$ . Satisfying the two conditions of $e^{T}J(\mu )e=0$ for all values of μ and $\left(R+R_{I}\right)>0$ , the system becomes exponentially and asymptotically stable at the equilibrium point [37].

Thus, the control rule given in equation (5.1) renders the system exponentially asymptotically stable with the damping injection.

Zero dynamics are defined as dynamics that characterize the internal behaviour of the system once the initial conditions and inputs are chosen such that the output is identically zero [38]. For stabilization through nonlinear PBC, we take into consideration the zero dynamics of the system to confirm the structural properties, in addition to the mathematical analysis mentioned above, to apply feedback control. The zero dynamics analysis of the boost converter as well as the buck-type inverter associated with the equilibrium points was given for the output as the capacitor voltage and the inductor current [28,32]. This analysis indicates that the dispersed generation network system is a non-minimum phase system with respect to the output voltage, but a minimum phase system with respect to the input inductor current. Thus, the chosen method of feedback is with the inductor current rather than the output voltage.

The nonlinear control equations are obtained from the exogenous control system given in equation (5.1). They play the role of a reference model with a stronger dissipation structure than the original system underscored by the added damping. The control equations are obtained separately for the AC ring, the DC rings and the connecting inverters as was done for the steady-state analysis. The control equations for the AC ring are given in equations (5.2)–(5.4)


(5.2) $$\begin{array}{c} \mu_{\alpha \text{n}}=\left[L_{\text{n}}{\overset{˙}{\bar{i}}}_{L\text{n}}+{\overset{˜}{v}}_{C\text{n}}\;-R_{\alpha \text{n}k}\left(i_{L\text{n}}-{\bar{i}}_{L\text{n}}\right)\right]/E_{\alpha \text{n}}, \end{array}$$

(5.3) $$\begin{array}{c} C_{\alpha \text{n}}{\overset{˙}{\overset{˜}{v}}}_{C\alpha \text{n}}={\bar{i}}_{L\alpha \text{n}}-{\overset{˜}{i}}_{T\alpha \text{n}}+{\overset{˜}{i}}_{T\alpha \text{n}-\text{1}}\\ -\left[\frac{{\overset{˜}{v}}_{C\alpha \text{n}}}{R_{2\alpha \text{n}-\text{1}}}\right]+\eta {\overset{˜}{i}}_{\alpha \beta }+\zeta {\overset{˜}{i}}_{\alpha \gamma }, \end{array}$$

(5.4) $$L_{T\alpha \text{n}}{\overset{˙}{\overset{˜}{i}}}_{T\alpha \text{n}}={\overset{˜}{v}}_{C\alpha \text{n}}-{\overset{˜}{v}}_{C\alpha \text{n}+\text{1}}-R_{1\alpha \text{n}}{\overset{˜}{i}}_{T\alpha \text{n}}.$$

Here, $x_{d\alpha \text{n}}={\left[{\bar{i}}_{L}\;\;{\tilde{v}}_{C}\;\;{\tilde{i}}_{LT}\right]}^{\text{T}}$ is the desired dynamic state vector of the controller for the inverters in the AC ring and $\mu_{\alpha \text{n}}$ is the feedback duty cycle. Next, the control equations for the inverters are obtained as follows:


(5.5) $$\begin{array}{c} \mu_{\text{inv}\beta }=\left[L_{\text{inv}\beta }{\overset{˙}{\bar{i}}}_{L\text{inv}\beta }+{\overset{˜}{v}}_{C\text{inv}\beta }\;-R_{\alpha \beta k}\left(i_{L\text{inv}\beta }-{\bar{i}}_{L\text{inv}\beta }\right)\right]/{\bar{v}}_{C\beta \text{q}}, \end{array}$$

(5.6) $$C_{\text{inv}\beta }{\overset{˙}{\overset{˜}{v}}}_{C\text{inv}\beta }={\bar{i}}_{L\text{inv}\beta }-\left[\frac{{\overset{˜}{v}}_{C\text{inv}\beta }}{R_{2\text{n}-\text{1}}}\right],$$

(5.7) $$L_{\alpha \beta }{\overset{˙}{\overset{˜}{i}}}_{T\alpha \beta }={\overset{˜}{v}}_{\text{inv}\beta }-{\overset{˜}{v}}_{C\alpha \text{n}}-R_{\alpha \beta }{\overset{˜}{i}}_{\alpha \beta }.$$

For the connecting inverter control equations, the DC input is given by the steady-state voltage of the corresponding DC/DC converter of the DC ring, in this case given by #q . The equations for the connecting inverter between γ−α rings can be obtained in a similar manner. Finally, the control rule for the DC rings is given in equations (5.8)–(5.10)


(5.8) $$\mu_{\beta \text{p}}=\frac{1}{{\overset{˜}{v}}_{C\beta \text{p}}}\left[E_{\beta \text{p}}+R_{\beta \text{p}k}\left(i_{L\beta \text{p}}-{\bar{i}}_{L\beta \text{p}}\right)\right]+1,$$

(5.9) $$\begin{array}{c} C_{\beta \text{p}}{\overset{˙}{\overset{˜}{v}}}_{C\beta \text{p}}=\left(1-\mu_{\beta \text{p}}\right){\bar{i}}_{L\beta \text{p}}-{\overset{˜}{i}}_{T\beta \text{p}}+{\overset{˜}{i}}_{T\beta \text{p}-\text{1}}-\left[\frac{{\overset{˜}{v}}_{C\beta \text{p}}}{R_{2\beta \text{p}-\text{1}}}\right]\\ -\mu_{\text{inv}\beta }{\bar{i}}_{\text{inv}\beta }, \end{array}$$

(5.10) $$L_{T\beta \text{p}}{\overset{˙}{\overset{˜}{i}}}_{T\beta \text{p}}={\overset{˜}{v}}_{C\beta \text{p}}-{\overset{˜}{v}}_{C\beta \text{p}+\text{1}}-R_{1\beta \text{p}}{\overset{˜}{i}}_{T\beta \text{p}}.$$

The constant desired inductor currents ${\bar{i}}_{L\alpha \text{n}}$ , ${\bar{i}}_{L\text{inv}\beta }$ , ${\bar{i}}_{L\text{inv}\gamma }$ , ${\bar{i}}_{L\beta \text{p}}$ and ${\bar{i}}_{L\gamma \text{s}}$ are obtained from the steady-state analysis. The state inductor current for all converter/inverter units is used for feedback control with corresponding damping $R_{\alpha \text{n}k}$ , $R_{\text{inv}\beta k}$ , $R_{\text{inv}\gamma k}$ , $R_{\beta \text{p}k}$ and $R_{\gamma \text{s}k}$ .

With the application of nonlinear PBC, the value of the duty ratio μ is calculated at every instant t with respect to the input, the system parameters and the desired state. That is, μ depends on time and the current state.

## 6. Numerical simulations

This section gives the numerical simulation results for a hybrid AC/DC system with five units in each ring. The numerical simulations were carried out on ode45 solver Simulink (Version 8.7 R2016a).

### 6.1. Simulation results for a balanced system

In this section, results are presented for numerical simulations performed for a balanced system with and without the application of PBC. A balanced state is when the parameters for all converters/inverters in each ring are identical, indicating the same values for the inductance, capacitance, load and dissipation between ( $L_{T}$ ) and the input voltages ( E ) are also set at the same value for each ring, respectively. This creates a natural balance within each ring, but not necessarily between the rings. These values are set as given in tables 1 [Table T2]–3 for α , β , γ rings and the connecting inverters.

**Table 1.** Parameters for α ring.

parameter	value	unit
$E_{\alpha \text{n}}$	36	V
$L_{\alpha \text{n}}$	46	mH
$C_{\alpha \text{n}}$	100	μF
$L_{T\alpha \text{n}}$	15	mH
$R_{1\alpha \text{n}}$	50	Ω
$R_{2\alpha \text{n}}$	100	Ω
${\bar{v}}_{C\alpha \text{n}}$ ( $A_{\text{n}}=13$ )	13sin⁡(376×t)	V

**Table 2. T2:** Connecting inverters.

parameter	value	unit
$L_{\text{inv}\beta }$	16	mH
$C_{\text{inv}\beta }$	100	μF
$R_{\text{inv}\beta }$	100	Ω
$R_{\alpha \beta }$	10	Ω
$L_{\alpha \beta }$	16	mH

**Table 3.** Parameters for β , γ ring.

parameter	value	unit
$E_{\beta \text{p}}$	18	V
$L_{\beta \text{p}}$	46	mH
$C_{\beta \text{p}}$	100	μF
$L_{T\beta \text{p}}$	15	mH
$R_{1\beta \text{p}}$	50	Ω
$R_{2\beta \text{p}}$	100	Ω
${\tilde{v}}_{C\beta \text{p}}$	40	V

**Figure 5.**
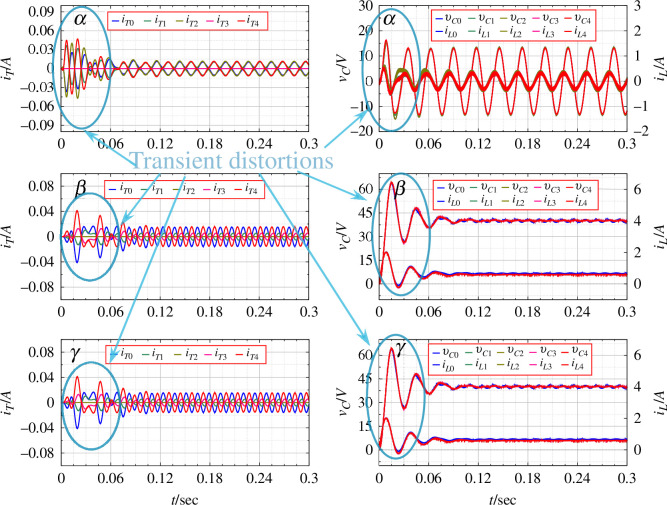
State variables for the α , β and γ rings for the original open-loop balanced system.

Figure 5 gives the result of numerical simulation for the distributed generation system without the application of PBC. Here, the duty ratios for the rings $U_{\alpha \text{n}}$ , $U_{\text{inv}\beta }$ , $U_{\text{inv}\gamma }$ , $U_{\beta \text{p}}$ and $U_{\gamma \text{s}}$ are set at a constant value estimated at the steady-state analysis. A disturbance in the load or the dissipation will not be controlled and will affect the system output adversely. It is seen that all three rings settle on the desired output voltage after the transient.

**Figure 6.**
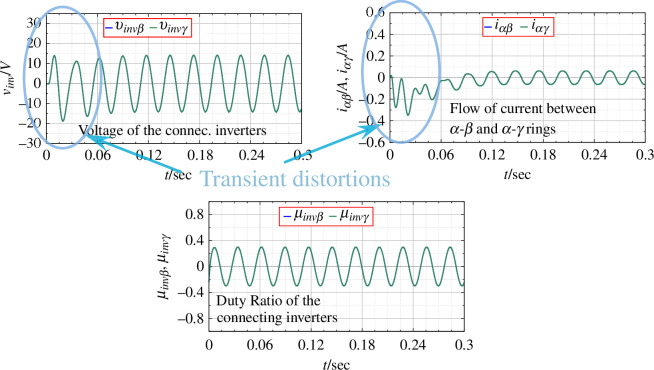
Parameters for connecting inverters for an open-loop balanced system.

The parameters for the β and γ rings have been set to be the same, creating symmetry and resulting in the output for the two DC rings to exactly equal. The duty ratio for the DC rings is constant, and sinusoidal for the α ring and the inverters as shown. The connection parameters for the inverters are given in figure 6. Even beyond the transient, it is seen that current flows between the converter/inverter units in the rings ( $i_{T\alpha \text{n}}$ , $i_{T\beta \text{p}}$ , $i_{T\gamma \text{s}}$ ) as well as between the α – β and α – γ rings as indicated by $i_{\alpha \beta }$ and $i_{\alpha \gamma }$ given in figure 5.

**Figure 7.**
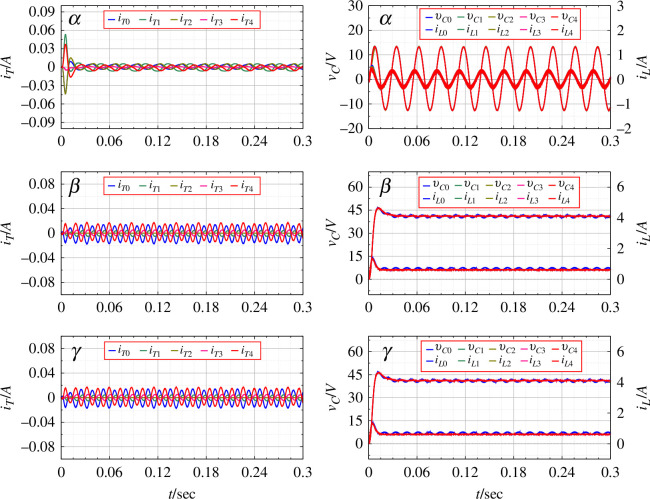
State variables for the α , β and γ rings with the application of PBC.

**Figure 8.**
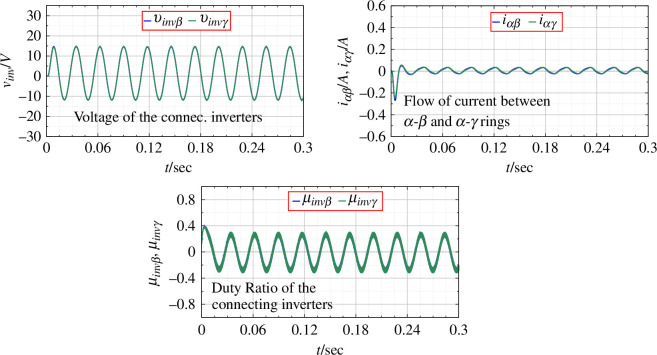
Parameters for connecting inverters with the application of PBC.

Next, the simulations with the application of PBC with the control rule in equation (5.1) are given. PBC is a nonlinear dynamic feedback controller that shapes the closed-loop energy by enhancing the closed-loop damping of the system. When adding the damping it is necessary that $R+R_{I}>0$ . The damping for the following numerical simulations was set at $R_{k}=20$ for all the converter/inverter units. Now the duty ratio is not constant, but a function of time as is shown, confirming the application of feedback control through passivity. The output voltage shows a faster convergence to the desired value with damped oscillations for the transient, as shown in figure 7. The flow of current through the rings ( $i_{T}$ ) as well as between the rings ( $i_{\alpha \beta }$ , $i_{\alpha \gamma }$ ) is reduced, allowing the system to attain stability by maintaining the passivity of the entire network. The connecting parameters for the inverters are given in figure 8.

### 6.2. Simulations for the system with disturbance

**Figure 9.**
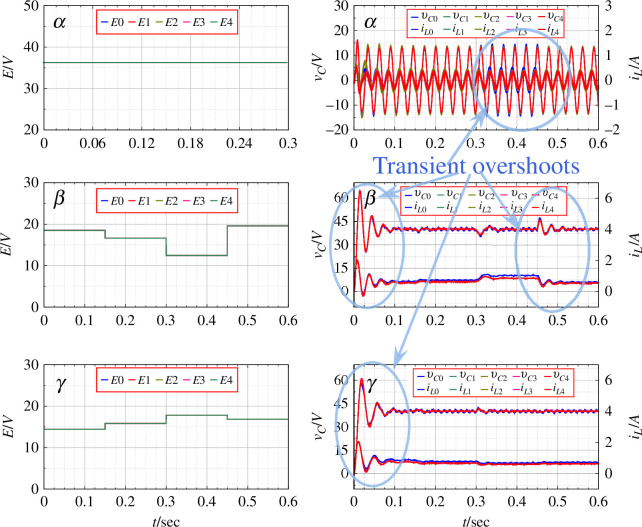
State variables for the α , β and γ rings for the original open-loop system with varying input voltage.

**Figure 10.**
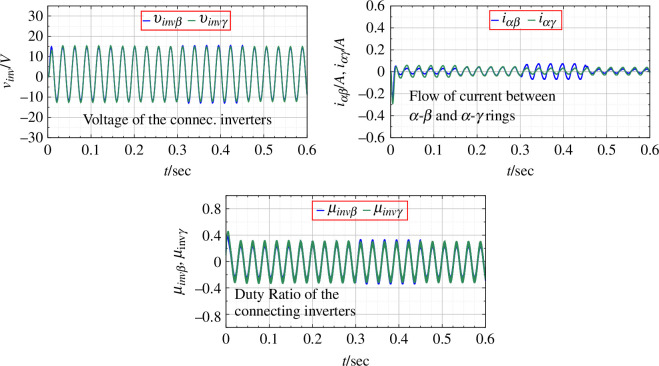
Parameters for connecting inverters for an open-loop system with varying input voltage.

If the inductance and capacitance values for the converter/inverter units or the dissipation between two neighbouring units are not symmetrical, it creates an imbalance in the ring. In this section, numerical simulations for systems with imbalance by varying input voltage values for the β and γ DC rings are considered. Practically, as the DC rings represent a distributed network in remote villages, it is assumed that the input voltage from the solar arrays might change depending on the weather conditions. The simulated change in input voltage and the corresponding output of the network are shown in figure 9. Here, PBC is not applied, and the output is based on the duty ratio calculated by the steady-state analysis. Figure 10 shows the connection parameters for the same. As the input varies, the system shows the transient from one state to another creating imbalance and a noticeable flow of current between the rings. The inverter output voltage for the β and γ rings varies according to the transient created by the changing input.

**Figure 11.**
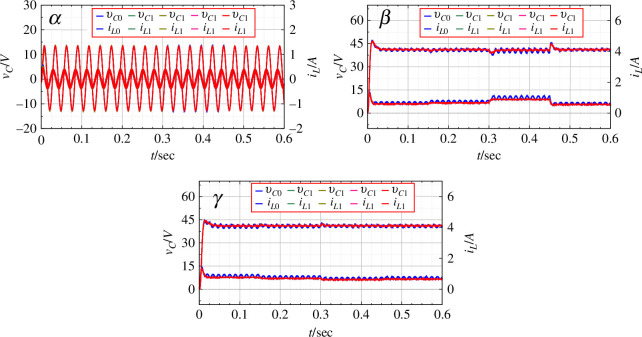
State variables for the α , β and γ rings with the application of PBC.

**Figure 12.**
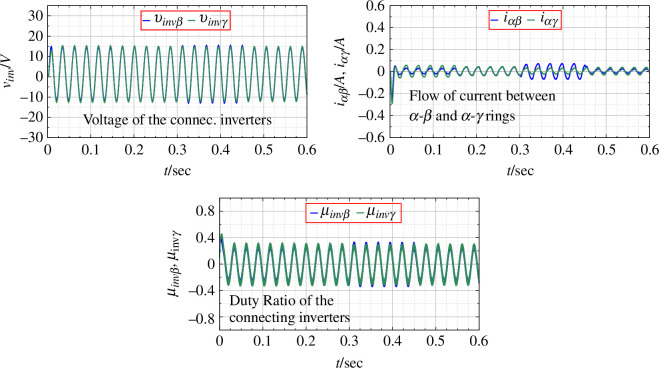
Parameters for connecting inverters with the application of PBC.

For the same change in input voltage, the application of PBC is shown in figure 11. The output voltage is seen to be devoid transient disturbances. The DC as well as the AC outputs are seen to be much closer to the desired values as compared to the original system. Figure 12 shows the duty ratio of connecting inverters, the output voltage and the flow of current between the rings as PBC is applied.

As passivity is invariant under negative feedback interconnection, the connection of two passive systems is a passive system. Thus, PBC is applied to the entire network by ensuring the passivity of individual converter/inverter units, ensuring the passivation of the whole network. The results allude to this fact. The flow of the line current through the ring at any point of time is zero, indicating that the entire system retains passivity. This is true even when there is flow of energy between individual converters/inverters.

## 7. Discussion and further research

The engineering of hybrid distributed generation systems needs to be consistent with a suitable and affordable extension of the network. For this reason, the inclusion of additional generation units as an integral part of the system model is desirable [2,39]. In the hybrid distributed generation network presented in this article, the numerical simulations have been carried out for a limited number of generation units per ring and a limited number of DC rings connected to the main AC ring. The extension of this model to a practical system applicable to small villages must be discussed. As is shown in equations (2.1)–(2.3), equations (2.4)–(2.6) and equations (2.7)–(2.9), the mathematical design facilitates the inclusion of further generator units. The same goes for the number of DC rings connected to the ‘main’ ring. The system design presented does not cause a loss of generality. The control equations have also been laid out to include an arbitrary number of units as well as DC rings.

The structure matrix J of the PCHM presents two highly desirable outcomes: not only is it necessary for system control, but can also act as an adjacency matrix for the analysis of vital system components and performances. The adjacency matrix (figure 3) reveals the dynamic connection between all nodes of the network at a glance. For expansion, it will be able to incorporate new node connections in the ring as well as jump connections between rings. Thus, the adjacency matrix provides valuable indicators of the distributed generation grid when expanded by adding a large number of units. Within this network representation framework, further analysis can be conducted to understand the robustness properties [40,41]. Here, robustness implies the ability of the distributed generation grid to be resilient to external disturbances, one of which is failure to operate smoothly subject to expansion [42]. The vulnerable nodes in the network can be detected, thus making expansion achievable with keeping the safety. Here, the numerical results are in congruence with the centrality analysis presented in §4. The degree centrality graph presented in figure 4 predicted the most significant nodes to be the voltages $v_{C\alpha \text{0}}$ , $v_{C\alpha \text{2}}$ , $v_{C\beta \text{0}}$ and $v_{C\gamma \text{0}}$ .

The aim of the network is to have multiple consumers connected together, with all units operating synchronously with equal output voltages. It is assumed that the consumers are scattered over a large area with varying distances between each other. The proposed hybrid network design considers the dissipation with a transmission line model. It enables the incorporation of variable dissipation parameters potentially determined by the distance between the units. PCHM with its well-ordered matrix structure allows effortless modification in the system parameters.

Based on the numerical simulations, it is clear that the AC as well as DC output with nonlinear PBC shows improved convergence time during transient operation. Nonlinear PBC takes into account the energy dynamics, and the physical structure of the original converter/inverter as well as its closed-loop formulation. In §5, it was mathematically proven that the entire network achieves Lyapunov stability with the application of nonlinear PBC. Moreover, the system converges exponentially to the desired state with the addition of damping. The numerical results show coherence with the analytical solutions. For the same input inductor and output capacitor values, the original system produces an under-damped transient response as opposed to the system with PBC, which shows a damped response.

Nonlinear controllers have been shown to be exceptionally good at trajectory tracking as compared to their linear counterparts [43,44]. It has been shown in comparative studies that dependence on linearization renders the system fragile, making it less attractive in applications [43]. With the nonlinear PBC, the exogenous system described in equations (5.2)–(5.10) is responsible for tracking the error based on the desired energy function. The controller anticipates the energy dynamics as well as the physical structure of the system, enhancing robustness and resilience to fluctuations during transient. The controller follows a time-variant desired state-trajectory created by the exogenous system forcing the duty ratio of each of the converter/inverter to change as a function of time. This is especially significant for the system transient response. As the nonlinear exogenous system modifies the duty ratio based on the dynamic energy structure to track the desired state trajectory, the system rapidly converges to the steady state, as shown in figure 7.

Power quality issues have been a challenge for distributed generation. For DC networks in the hybrid microgrid, these can correspond to voltage drops, insufficient power input from one or more of the distributed generation units and sudden load changes. For AC networks in the hybrid microgrid, these may correspond to harmonic distortion, abrupt frequency changes and phase unbalance. A well-described PCHM framework and a well-suited energy function for deriving the PBC equations can address these issues. Depending on the chosen energy function, stability can be guaranteed and the design of the energy function can make it possible for the system to respond to sudden changes in the load. This is confirmed from the numerical results of the original system (without control) when the input voltage is varied, as shown in figure 9. For an unbalanced system, the states of the connecting converters $\alpha_{\text{0}}$ , $\alpha_{\text{2}}$ , $\beta_{\text{0}}$ and $\gamma_{\text{0}}$ are the only ones that vary from the desired trajectory. For the same conditions, PBC exhibits accelerated convergence to the desired state.

As each unit (either a converter or an inverter) in distributed generation needs its own control input, another challenge faced by distributed generation is the effect of time delays and noise. For smooth voltage and frequency control, it is imperative that the control should be robust to time delays. For the control method suggested in this article, this problem is partially addressed by the method for calculation of the control input. With PCHM, the system is neatly defined in matrices, which renders it easy to solve meaning that the calculation time is greatly reduced as compared with similar nonlinear methods. To allow for enhanced noise reduction, it is possible to adjust the energy function such that the control equations in turn are modified to include active filtering properties. To the best of our knowledge, such techniques are scarcely considered in literature with the notable exceptions being [45] in the context of fuel cells.

Another possible area of further research for nonlinear PBC with an expanding distributed generation network is through reinforcement learning. Reinforcement learning is an area of machine learning where the optimal behaviour is learned in a given environment to obtain the maximum reward. In the context of nonlinear PBC, based on the given structure of the AC/DC hybrid microgrid, reinforcement learning can be used to obtain the best suitable energy function to apply for a faster control action. Simulation results from the given structure can be used to learn a more optimal definition of an energy function.

## 8. Conclusion

In this article, a nonlinear PBC technique was introduced to provide voltage control for the AC and DC sides of a stand-alone hybrid microgrid. To this end, first of all, a network framework for the structure of a hybrid AC/DC microgrid was developed. The framework considers multiple DC grids connected to a central single-phase AC microgrid. A mathematical modelling of this network framework was proposed with the PCHM approach. The main contribution of this article was proposing a nonlinear control technique to control all the converters and inverters in the proposed hybrid microgrid to obtain voltage control for the DC network of the hybrid microgrid and voltage control along with phase and frequency synchronization for the AC network of the microgrid.

Nonlinear PBC is a control method that shapes an energy function to have a stable equilibrium at the desired state, implemented by using a quadratic function, and impedance damping was added to enhance the asymptotic stability. To derive the equations for nonlinear PBC, first, steady-state analysis is conducted to find the constant duty ratio to attain the desired voltage for the DC as well as AC networks in the steady state. The steady-state representation provides a default control algorithm and can be used in the event of time delays and unforeseen circumstances where the control may be unavailable.

A quadratic energy function based on the total energy of the circuit was chosen as the cost function to design the nonlinear control equations for PBC. It was shown that the system is asymptotically stable with the application of the control equations, and impedance damping was added for exponential stability. It was shown that by minimizing the desired storage function, it was possible to attain the user-defined desired output voltage by regulation of the duty cycle through feedback of the inductor current of the multiple power electronic inverters/converters in the network.

Numerical simulations were performed to confirm the application of the control method in the transient as well as the steady state. Numerical simulations were performed for a balanced case as well as under varying inputs to the network. The state variables of inductor current and capacitor output voltage as well as the current through the dissipation within each ring component of the network were obtained. The flow of current between the rings and the operation of the connecting inverters was investigated for both the original system as well as feedback control through PBC. The results indicate that the application of PBC significantly improves the system response time and drives the system to a stable equilibrium in an exponential time.

## Data Availability

Data and relevant code for this research work are stored in GitHub [46] and have been archived within the Zenodo repository [47]. The details of the Matlab simulation file are provided here. The conditions were changed to obtain all the results presented in this article.

## References

[1] Pepermans G , Driesen J , Haeseldonckx D , Belmans R , D’haeseleer W . 2005 Distributed generation: definition, benefits and issues. Energy Policy **33** , 787–798. (10.1016/j.enpol.2003.10.004)

[2] Ackermann T , Andersson G , Söder L . 2001 Distributed generation: a definition. Elec. Power Syst. Res. **57** , 195–204. (10.1016/S0378-7796(01)00101-8)

[3] El-Khattam W , Salama MMA . 2004 Distributed generation technologies, definitions and benefits. Elec. Power Syst. Res. **71** , 119–128. (10.1016/j.epsr.2004.01.006)

[4] Mitra I , Degner T , Braun M . 2008 Distributed generation and microgrids for small island electrification in developing countries: a review. SESI **18** , 6–20.

[5] Bhoyar R , Bharatkar S . 2012 Potential of microsources, renewable energy sources and application of microgrids in rural areas of Maharashtra state India. Energy Procedia **14** , 2012–2018. (10.1016/j.egypro.2011.12.1202)

[6] Beck F , Martinot EE . 2004 Renewable energy policies and barriers. In Encyclopedia of energy, vol. 5, pp. 365–383. Amsterdam, The Netherlands: Elsevier.

[7] Nair NKC , Zhang L . 2009 Smartgrid: future networks for New Zealand power systems incorporating distributed generation. Energy Policy **37** , 3418–3427. (10.1016/j.enpol.2009.03.025)

[8] Banerjee B , Islam SM . 2011 Reliability based optimum location of distributed generation. Int. J. Electr. Power Energy Syst. **33** , 1470–1478. (10.1016/j.ijepes.2011.06.029)

[9] Chauhan A , Saini R . 2015 Renewable energy based off-grid rural electrification in Uttarakhand state of India. Ren. Sust. Energy Rev. **51** , 662–681. (10.1016/j.rser.2015.06.043)

[10] Castellanos JG , Walker M , Poggio D , Pourkashanian M , Nimmo W . 2015 Modelling an off-grid integrated renewable energy system for rural electrification in India using photovoltaics and anaerobic digestion. Ren. Energy **74** , 390–398. (10.1016/j.renene.2014.08.055)

[11] Agarwal N , Kumar A . 2013 Optimization of grid independent hybrid PV–diesel–battery system for power generation in remote villages of Uttar Pradesh, India. Energy Sustain. Dev. **17** , 210–219. (10.1016/j.esd.2013.02.002)

[12] Sen R , Bhattacharyya SC . 2014 Off-grid electricity generation with renewable energy technologies in India: an application of HOMER. Ren. Energy **62** , 388–398. (10.1016/j.renene.2013.07.028)

[13] Jia L , Zhu Y , Wang Y . 2015 Architecture design for new AC-DC hybrid micro-grid. In 2015 IEEE 1st Int. Conf. on DC Microgrids (ICDCM), *Atlanta, GA, USA, 7–10 June 2015* . (10.1109/ICDCM.2015.7152020)

[14] Nejabatkhah F , Li YW . 2015 Overview of power management strategies of hybrid AC/DC microgrid. IEEE Trans. Power Electron. **30** , 7072–7089. (10.1109/TPEL.2014.2384999)

[15] Loh PC , Li D , Chai YK , Blaabjerg F . 2013 Autonomous control of interlinking converter with energy storage in hybrid AC–DC microgrid. IEEE Trans. Ind. Appl. **49** , 1374–1382. (10.1109/TIA.2013.2252319)

[16] Nasir M , Khan HA , Hussain A , Mateen L , Zaffar NA . 2018 Solar PV-based scalable DC microgrid for rural electrification in developing regions. IEEE Trans. Sustain. Energy **9** , 390–399. (10.1109/TSTE.2017.2736160)

[17] Khodayar ME . 2017 Rural electrification and expansion planning of off-grid microgrids. Electr. J. **30** , 68–74. (10.1016/j.tej.2017.04.004)

[18] Popov VM , Georgescu R . 1973 Hyperstability of control systems. Berlin, Germany: Editura Academiei, Springer-Verlag. (10.1007/978-3-642-65654-5)

[19] Ortega R , Perez JAL , Nicklasson PJ , Sira-Ramirez H . 2013 Passivity-based control of Euler-Lagrange systems: mechanical, electrical and electromechanical applications. London, UK: Springer Science & Business Media.

[20] Ortega R , Van Der AJ , Mareels I , Maschke B . 2001 Putting energy back in control. IEEE Control Syst. **21** , 18–33. (10.1109/37.915398)

[21] Murillo-Yarce D , Garcés-Ruiz A , Escobar-Mejía A . 2018 Passivity-based control for DC-microgrids with constant power terminals in island mode operation. Rev. Fac. Ing. Univ. Antioq. 32–39. (10.17533/udea.redin.n86a05)

[22] Hassan MA , He Y . 2020 Constant power load stabilization in DC microgrid systems using passivity-based control with nonlinear disturbance observer. IEEE Access **8** , 92393–92406. (10.1109/ACCESS.2020.2992780)

[23] Hassan MA , Su CL , Chen FZ , Lo KY . 2021 Adaptive passivity-based control of a DC–DC boost power converter supplying constant power and constant voltage loads. IEEE Trans. Ind. Electron. **69** , 6204–6214. (10.1109/TIE.2021.3086723)

[24] He W , Namazi MM , Koofigar HR , Amirian MA , Blaabjerg F . 2021 Stabilization of DC–DC buck converter with unknown constant power load via passivity‐based control plus proportion‐integration. IET Power Electron. **14** , 2597–2609. (10.1049/pel2.12205)

[25] Hassan MA , Li E ping , Li X , Li T , Duan C , Chi S . 2018 Adaptive passivity-based control of DC–DC buck power converter with constant power load in DC microgrid systems. IEEE J. Emerg. Sel. Topics Power Electron. **7** , 2029–2040. (10.1109/JESTPE.2018.2874449)

[26] Samanta S , Mishra S , Ghosh T . 2022 Control of DC microgrid using interconnection and damping assignment passivity based control method. In IEEE 7th Int. Conf. for Convergence in Technology (I2CT), Mumbai, India, 7–9 April 2022, pp. 1–6. (10.1109/I2CT54291.2022.9824092)

[27] Gui Y , Wei B , Li M , Guerrero JM , Vasquez JC . 2018 Passivity-based coordinated control for islanded AC microgrid. Appl. Energy **229** , 551–561. (10.1016/j.apenergy.2018.07.115)

[28] Manohar R , Hikihara T . 2020 Phase synchronization of autonomous AC grid system with passivity‐based control. Int. J. Circuit Theory Appl. **48** , 906–918. (10.1002/cta.2760)

[29] Amirkhan S , Radmehr M , Rezanejad M , Khormali S . 2019 An improved passivity-based control strategy for providing an accurate coordination in a AC/DC hybrid microgrid. J. Franklin Inst. **356** , 6875–6898. (10.1016/j.jfranklin.2019.03.026)

[30] Azimi SM , Hamzeh M . 2020 Adaptive interconnection and damping assignment passivity-based control of interlinking converter in hybrid AC/DC grids. IEEE Syst. J. **14** , 4718–4725. (10.1109/JSYST.2019.2961314)

[31] Ortega R , van der Schaft A , Maschke B , Escobar G . 1999 Energy-shaping of port-controlled Hamiltonian systems by interconnection. In Proc. 38th IEEE Conf. on Decision and Control, Phoenix, AZ, USA, 7–10 December 1999, pp. 1646–1651. (10.1109/CDC.1999.830260)

[32] Manohar R , Hikihara T . 2020 Dynamic behaviour of a ring coupled boost converter system with passivity-based control. NOLTA **11** , 109–122. (10.1587/nolta.11.109)

[33] Maschke BM , van der Schaft AJ . 1992 Port-controlled Hamiltonian systems: modelling origins and systemtheoretic properties. IFAC Proc. Vol. **25** , 359–365. (10.1016/S1474-6670(17)52308-3)

[34] van der Schaft AJ . 2004 Port-Hamiltonian systems: network modeling and control of nonlinear physical systems. In Advanced dynamics and control of structures and machines (eds H Irschik , K Schlacher ), pp. 127–167. Vienna, Austria: Springer. (10.1007/978-3-7091-2774-2_9)

[35] Pósfai M , Barabási AL . 2016 Network science. Cambridge, UK: Cambridge University Press.

[36] Pagani GA , Aiello M . 2013 The power gridas a complex network: a survey. Phys. A Stat. Mech. Appl. **392** , 2688–2700. (10.1016/j.physa.2013.01.023)

[37] Khalil HK , Grizzle J . 1996 Nonlinear systems, vol. 3. Upper Saddle River, NJ: Prentice Hall.

[38] Isidori A . 2013 The zero dynamics of a nonlinear system: from the origin to the latest progresses of a long successful story. Eur. J. Control **19** , 369–378. (10.1016/j.ejcon.2013.05.014)

[39] Zangeneh A , Jadid S , Rahimi-Kian A . 2010 Uncertainty based distributed generation expansion planning in electricity markets. Electr. Eng. **91** , 369–382. (10.1007/s00202-010-0146-6)

[40] Mao Y , Liu F , Mei S . 2010 On the topological characteristics of power grids with distributed generation. In Proc. 29th Chinese Control Conf., Beijing, China, 29–31 July 2010, pp. 4714–4720.

[41] Liu C , Xu Q , Chen Z , Bak CL . 2012 Vulnerability evaluation of power system integrated with large-scale distributed generation based on complex network theory. In 2012 47th Int. Universities Power Engineering Conf., London, UK, 4–7 September 2012, pp. 1–5. (10.1109/UPEC.2012.6398605)

[42] Cuadra L , Salcedo-Sanz S , Del Ser J , Jiménez-Fernández S , Geem ZW . 2015 A critical review of robustness in power grids using complex networks concepts. Energies **8** , 9211–9265. (10.3390/en8099211)

[43] Kim KC , Ortega R , Charara A , Vilain JP . 1997 Theoretical and experimental comparison of two nonlinear controllers for current-fed induction motors. IEEE Trans. Contr. Syst. Technol. **5** , 338–348. (10.1109/87.572130)

[44] Rodriguez H , van der Schaft AJ , Ortega R . 2001 On stabilization of nonlinear distributed parameter port-controlled Hamiltonian systems via energy shaping. In Proc. 40th IEEE Conf. on Decision and Control, *Orlando, FL, USA, 4–7 December 2001* , pp. 131–136. (10.1109/CDC.2001.980086)

[45] Hilairet M , Ghanes M , Béthoux O , Tanasa V , Barbot JP , Normand-Cyrot D . 2013 A passivity-based controller for coordination of converters in a fuel cell system. Control Eng. Pract. **21** , 1097–1109. (10.1016/j.conengprac.2013.04.003)

[46] Manohar R . MATLAB. See https://github.com/rutvikam/distributedgeneration_hybridmicrogrid.

[47] Manohar R . 2024 rutvikam/distributedgeneration_hybridmicrogrid: distributedgeneration_hybridmicrogrid_ver2 (Version ver2). Zenodo. See 10.5281/zenodo.11000609.

